# An enzymatic on/off switch‐mediated assay for *KRAS* hotspot point mutation detection of circulating tumor DNA

**DOI:** 10.1002/jcla.23305

**Published:** 2020-03-24

**Authors:** Qing‐lin Wang, Cui‐lan Zhou, Yu‐fang Yin, Li Xiao, Yuan Wang, Kai Li

**Affiliations:** ^1^ Jiangsu Key Laboratory of Neuropsychiatric Diseases and College of Pharmaceutical Sciences Soochow University Suzhou China; ^2^ Department of Human Anatomy University of South China Hengyang China; ^3^ Department of Pharmacology and Neuroscience SIU Medical School Springfield IL USA; ^4^ Laboratory of Molecular Medicine The Second Affiliated Hospital of Soochow University Suzhou China; ^5^ GeneTalks Biotechnology Inc. Changsha China

**Keywords:** ctDNA, enzymatic on/off switch, *KRAS* mutations, multiplex PCR, mutation detection

## Abstract

**Background:**

To detect the mutations of *KRAS* gene in colorectal cancer patients and other cancer patients, it is of value to develop non‐invasive, sensitive, specific, easy, and low‐cost assays.

**Methods:**

Templates harboring hotspot mutations of the *KRAS* gene were constructed, and primers were designed for evaluation of the specificity, and sensitivity of detection system consisted of exonuclease polymerase‐mediated on/off switch; then, gel electrophoresis and real‐time PCR were performed for verification. The assay was verified by testing the DNA pool of normal controls and circulating DNA (ctDNA) samples from 14 tumor patients, as compared to Sanger sequencing.

**Results:**

A specific and sensitive assay consisted of exonuclease polymerase‐mediated on/off switch, and multiplex real‐time PCR method has been established. This assay could detect <100 copies of *KRAS* mutation in more than 10 million copies of wild‐type *KRAS* gene fragments. This assay was applied to test *KRAS* gene mutations in three cases of fourteen ctDNA samples, and the results were consistent with Sanger sequencing. However, this PCR‐based assay was more sensitive and easier to be interpreted.

**Conclusion:**

This assay can detect the presence of *KRAS* hotspot mutations in clinical circulating tumor DNA samples. The assay has a potential to be used in early diagnosis of colorectal cancer as well as other types of cancer.

## 1 INTRODUCTION

It is widely accepted that accumulation of mutations and chromosomal aberrations is the major causes of cancers.^1^ Mutations in *KRAS* are detected in approximately 30% of all human cancers.^2^ KRAS is involved in intracellular signal transduction, such as epidermal growth factor receptor (EGFR) signaling pathway.^3^ KRAS is activated abnormally when the *KRAS* gene is mutated.^4^ Point mutation rate of *KRAS* gene is 40%‐50% in colorectal cancer. The mutations are mainly located in codons 12 and 13 with approximate 80% occurrence in the 12th codon, 15% occurrence in the 13th codon, and 5% occurrence in codon 61 and others.^5^ The most majority of *KRAS* mutations identified are single nucleotide point mutations. The common patterns include G12D, G12A, G12R, G12C, G12S, G12V, and G13D. The mutation pattern of *KRAS* provides guidance of individualized treatment, and the *KRAS* wild‐type colorectal cancer patients can benefit from treatment with anti‐epidermal growth factor cetuximab.^6^, ^7^ *KRAS* mutation identification is strongly recommended by FDA to guide usage of cetuximab. Therefore, a sensitive, specific, and convenient method with detection capability of multi‐mutations is highly desirable. In the past several decades, Sanger sequencing has been served as golden standards in the field.^8^, ^9^ Sanger sequencing interrogates every base and identifies known and unknown variants, yet it requires post‐PCR manipulations such as cleanup of PCR. Another more critical disadvantage is its low detection sensitivity. It requires an abundance of 15% or more of a somatic DNA variant to be detected, which may lead to a false‐negative result in some cases.^8^, ^9^, ^10^, ^11^ Allele‐specific real‐time PCR identifies *KRAS* mutations are sensitive enough but prone to false positive because of taq DNA polymerase.^9^, ^11^, ^12^, ^13^ High‐resolution melting analysis is the quantitative analysis of PCR products melting curve at different temperatures. Whereas it is highly sensitive and requires no post‐PCR process, melting curve analysis has no multiplex capacity and the result is difficult to be interpreted.^14^, ^15^ SnaPShot and next‐generation sequencing, the sensitivity of SnaPshot for *KRAS* mutation detection was about 10%, but this method is also laborious and time‐consuming.^16^, ^17^ Next‐generation sequencing is relatively expensive, time‐consuming, and complicated data interpreting.^18^, ^19^ Choong et al detected the *KRAS* mutations using the isothermal‐based optical sensor for companion diagnostics,^20^ a rapid, specific, and sensitive method, but the preparation process is relatively complex and the result is not easy to interpret. Therefore, a high‐throughput, economical, sensitive, and easy data analysis assay is urgently needed. Mutation‐sensitive on/off switch,^21^, ^22^ consisting of high‐fidelity DNA polymerase and phosphorothioate‐modified allele‐specific primers, has been established to have a higher specificity compared with assays mediated by low‐fidelity DNA polymerase. Table 1 shows the comparison of the *KRAS* mutation detection assay.

**Table 1:** Comparison of the *KRAS* mutation detection assays

Assay	Specificity	Sensitivity	Multiplex	Time‐saving	Result analysis
Multiplex PCR of on/off switch	Good	Good	Yes	Yes	Easy
Sanger sequence	Good	Ordinary	Yes	No	Difficult
HRM	Good	Good	No	Yes	Difficult
Allele‐specific real‐time PCR	Ordinary	Good	Yes	Yes	Easy
Isothermal‐based optical sensor	Good	Good	No	Yes	Difficult

Cell‐free DNA (cfDNA) is defined as extracellular DNA dissociating in the circulating system. The consistency of DNA alterations between a tumor and cfDNA proves the tumoral origin of the cfDNA.^23^ Circulating tumor DNA is derived from the release of nucleic acids during the apoptosis and necrosis of tumor cells or from tumor‐derived exosomes.^24^ In cancer patients, ctDNA presents as a variable fraction of cfDNA (ranging from <0.01% to more than 50%).^25^ The lengths of ctDNA from tumor patients range from 200 nt to longer than 1 kb.^26^ As biomarkers of cancer,^27^ the qualitative and quantitative analyses of the ctDNA in the blood have been used for early diagnosis of cancer, individualized therapy, prognosis, etc A highly sensitive, specific method might be established for clinical diagnostics base on ctDNA samples. Here, we developed an assay of detecting *KRAS* hotspot mutations in ctDNA. Our assay provides a new approach for the detection of somatic mutations such as *KRAS* mutations. This mutation identification strategy is based on the molecular switch consisting of high‐fidelity enzymes and the 3‐terminal phosphorylation‐modified primer. The mutation detection primers can only be extended when it perfectly matches the template. When primer and template are not perfectly matched, no or very low amount amplified products will be yielded, since high‐fidelity enzymes cannot remove mismatched base due to its phosphorothioate modification. This assay can identify *KRAS* mutations in a normal background from an minimal amount ctDNA of peripheral blood. This assay is low‐cost, fast, and high‐throughput with 96‐well or 384‐well plates for thousands of samples per day. It may serve as a non‐invasive and efficient diagnostic project for cancer patients or high‐risk individuals.

## 2 MATERIALS AND METHODS

### 2.1 The Statistical analysis of the database

The gene mutation data and survival data of the colorectal cancer patients were downloaded from The Cancer Genome Atlas (TCGA) database. The overall survival rates between patients with *KRAS* mutations and patients without *KRAS* mutations were analyzed by Kaplan‐Meier, and *P* < .05 was considered statistically significant.

### 2.2 Preparation of *KRAS* hotspot mutation templates

Preparation *of KRAS* hotspot mutation templates *was carried out* by the method of overlapping extension PCR*.* Sixteen primers were listed in Table S1. These primers were designed according to the reference sequences of Gene ID 3845 in Genbank. The primers were designed to introduce hotspots mutations at codon 12 and codon 13 of *KRAS* gene. The primer‐extended products were purified again and then subcloned into PGEM‐T Easy Vector (Invitrogen). Subcloned products were sequenced for confirmation. The seven mutation templates of *KRAS* gene, including G12D, G12V, G12S, G12C, G12R, G12A, and G13D, are shown in Figure S1.

### 2.3 The condition of the detection of *KRAS* mutations

The following nine mutation‐specific primers were designed for mutation analysis (Table 2). The RKRAS is the common reverse primer. All primers were synthesized by Invitrogen, and the bold underlined nucleotide was phosphorothioate modified. A single PCR or multiplex PCR with proofreading polymerase‐mediated on/off switch for *KRAS* mutations were detected in an ABI Prism 7500 system (Applied Biosystems). A 25 µL PCR detection system contained 2.5 µL of 10× Pfu buffer with MgSO_4_ (Fermentas), 1.2 µL of 20× Eva green (Biotium), 5n µmol/L of RKRAS primer, 5n µmol/L of FKRAS (n is depending on the number of primer), 0.5 U of Pfu, 0.4 µm of dNTP, 1 μl of template DNA, and ddH_2_O (to make up to 25 μl in total volume). After denature at 95°C for 5 minutes, primer extension was performed for 45 cycles with gradient PCR program, and the first stage was performed 10 cycles as denaturing at 95°C for 20 seconds, annealing at 68°C for 20 seconds with each cycle decreasing 1°C, and extension at 72°C for 30 seconds and the second stage 23 cycles, denaturing at 95°C for 20 seconds, annealing at 59°C and extension at 72°C for 30 seconds, and a final extension at 72°C for 10 minutes.

**Table 2:** The phosphorothioate‐modified primers for detecting the seven hotspot mutations of KRAS gene

FKRAS12‐1	5′‐TAAACTTGTGGTAGTTGGAGCTG †**A**† TC‐3′
FKRAS12‐2	5′‐TAAACTTGTGGTAGTTGGAGCTG †**T**†TC‐3′
FKRAS12‐3	5′‐TAAACTTGTGGTAGTTGGAGCT †**A**†GC‐3′
FKRAS12‐4	5′‐TAAACTTGTGGTAGTTGGAGCT † **T**†GC‐3′
FKRAS12‐5	5′‐TAAACTTGTGGTAGTTGGAGCT † **C**†GC‐3′
FKRAS12‐6	5′‐TAAACTTGTGGTAGTTGGAGCT G†**C**† TC‐3′
FKRAS13	5′‐TTGTGGTAGTTGGAGCTGGTG † **A**†CA‐3′
FK‐12	5′‐TAAACTTGTGGTAGTTGGAGCTG†**G**†TC‐3′
FK‐13	5′‐TTGTGGTAGTTGGAGCTGGTG†**G**†CA‐3′
RKRAS	5′‐TCTGTATCAAAGAATGGTCCTGC‐3′

Note

Where "†" in place of phosphorothioate modified, base which is marked in bold and underlined meaning mutant base.

### 2.4 PCR conditions for amplifying targets for *KRAS* mutations sequencing analysis

Taq polymerase‐mediated regular PCR was used for sequencing of the DNA samples from the normal controls and cancer patients. The primers for amplifying the products for sequencing are listed in Table S1. This regular PCR was performed in a volume of 25 µL system containing 12.5 µL of 2× mix (Biomega), 10 µmol/L of primers, and about 40 ng of genomic DNA. After denature at 95°C for 5 minutes, primer extension was performed for 30 cycles with denaturing at 95°C for 20 seconds, annealing at 55°C for 20 seconds, extension at 72°C for 30 seconds, and with a final extension at 72°C for 10 minutes.

### 2.5 Genomic DNA samples and ctDNA samples preparation

One pooled DNA sample contained two hundred normal controls was provided by the Nanhua University First Affiliated Hospital. Almost, the same amount of DNA in different final volume of each sample was mixed to form the gene pool after adjusted their concentrations of individual DNA samples.

Fourteen blood samples of tumor patients from the Second Affiliated Hospital of Soochow University were collected. Two mL of blood from the cubital vein was drawn to EDTA tubes and centrifuged at 12 000 *g* for 2 minutes, the ctDNA was extracted with the circulating DNA Extraction Kit (Omega), and extracted ctDNA was quantitatively measured by UV spectrometry.

## 3 RESULTS

### 3.1 The prognosis of colorectal cancer patients with *KRAS* mutations was more poor compared with those without mutations

**Figure 1:**
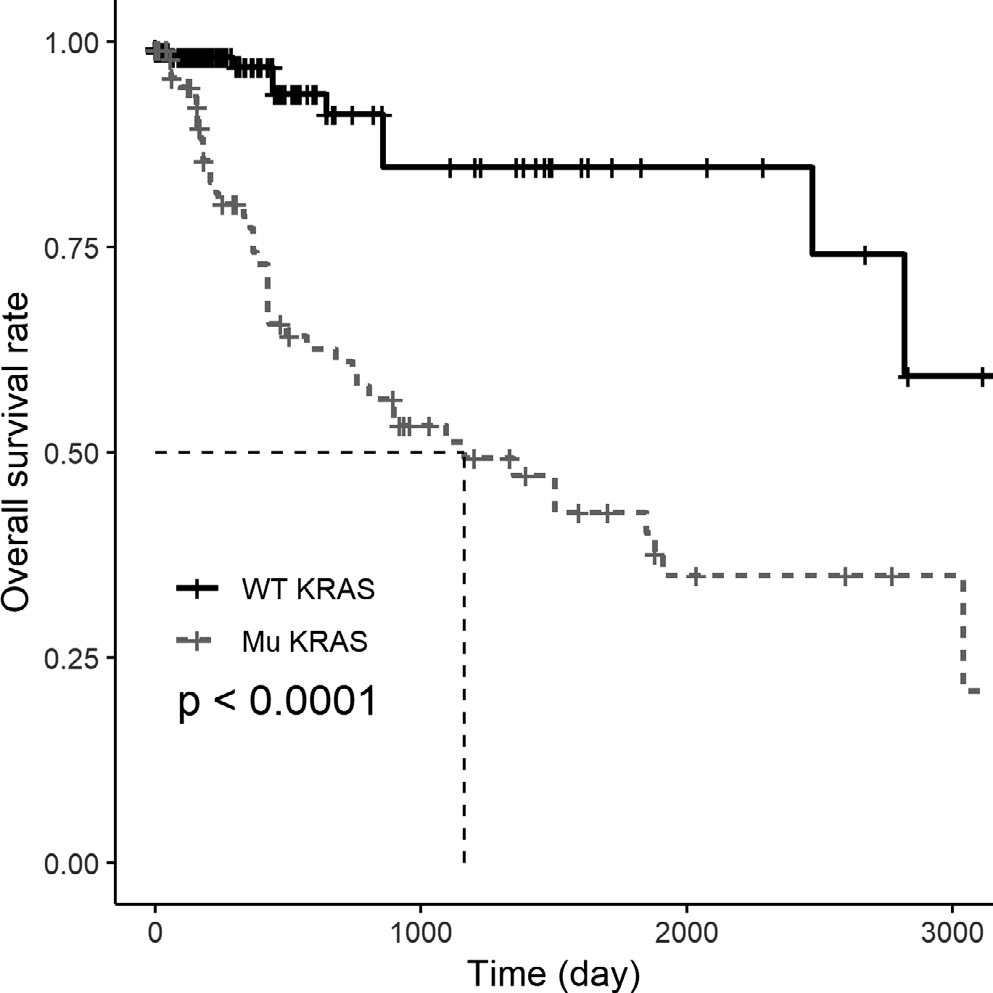
The analysis of overall survival rate according to *KRAS* status in colorectal cancer patients. Colorectal cancer patients with *KRAS* mutations have significantly shorter survival time than patients with wild‐type *KRAS*. (*P* < .0001)

Taking advantage of The Cancer Genome Atlas (TCGA) database, the pattern of *KRAS* mutations in colorectal cancer patients was analyzed (Data S1, download from https://portal.gdc.cancer.gov/). A total of 103 *KRAS* mutations have been found in 263 cancer patients. The total survival rate of colorectal cancer patients harboring *KRAS* mutations was significantly lower than that of the patients without *KRAS* mutations (Figure 1). Therefore, it is of great clinical value of differentiating *KRAS* mutations to provide guidance of individualized treatments of colorectal cancer patients.

### 3.2 A PCR‐based assay of *KRAS* mutation detection was established

**Figure 2:**
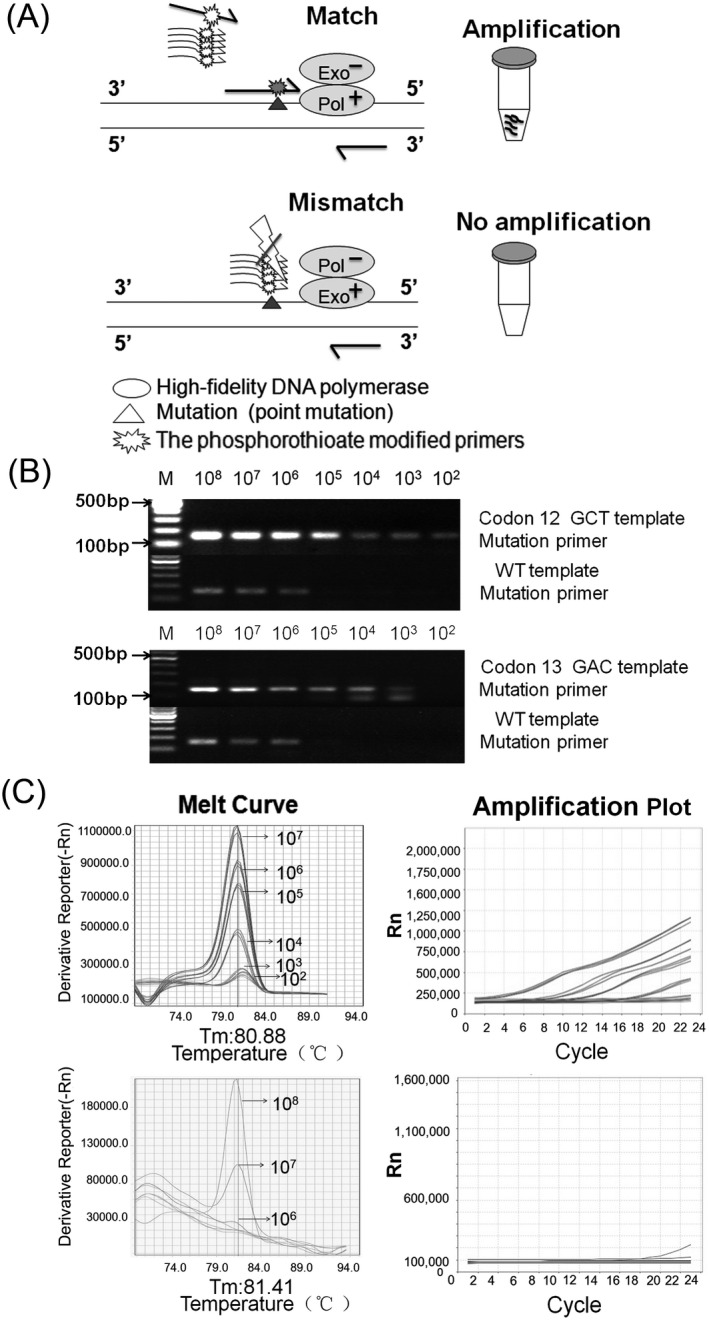
The assay establishment for the *KRAS* mutation detection mediated by an enzymatic on/off switch. A, An illustration showing the multiplex PCR experimental design by on/off switch. B, Taking the 12GCT and 13GAC of *KRAS* as examples, the mutant template and Wt template were diluted in 10‐fold serial and amplified by PCR with mutation‐specific primer. The length of the PCR products was both 155 bp. C, Establishment of a multiplex PCR system with codon 12, as the melt curve and amplification plot shows, the detection limit reach to 10^2^ copies, and the specificity can reach to 10^6^

We established the detection assays of single or multiplex PCR system combination with high‐fidelity enzyme “on/off”switch. As shown in Figure 2A, the multiplex PCR experimental design enables one of the codon 12 mutation primers to amplify the matched mutation templates. No observable products yielded from the mismatched primers. The seven mutant templates and the wild‐type template were diluted to a final concentration ranging from 10^8^ to 10^2^ copies per PCR reaction (25 µL) to test the sensitivity and specificity of the assay. The specificity and sensitivity of the seven primer pairs were finally determined by electrophoresis, false‐positive primer extension in most of the seven primer pairs was not produced until copy number of the mismatched template reached 10^7^, and few of them reached 10^6^. The sensitivity of the six mutation‐specific primers for codon 12 of *KRAS* was at least 10^2^ copies, and the codon 13 of *KRAS* was as low as 10^3^ copies (Figure 2B). The codon 13 mutation primer can yield a dimer while the template concentration is low. The codon 12GCT and codon 13GAC were illustrated as representatives are shown in Figure 2B. Figure 2C shows that real‐time PCR is more sensitive than conventional PCR. The sensitivity of this assay by real‐time PCR for the *KRAS* seven hotpot mutations is at least 10 times higher than conventional PCR without compromising its specificity of this assay. Considering the high similarity in DNA sequence of primers to target against the 6 nucleotide variants at the mutation hotspot of codon 12 of *KRAS* gene, we developed multiplex PCR of codon 12. The sensitivity of all primers could reach at least 10^2^ copies, while false‐positive amplification was not observed when the copy number of mismatched template was below 10^6^ copies. The final appropriate concentration of clinical samples in this assay is about between 10^2^ and 10^5^ copies, and this range is safe for clinical testing.

### 3.3 *KRAS* mutations cannot be detected in healthy human DNA samples

**Figure 3:**
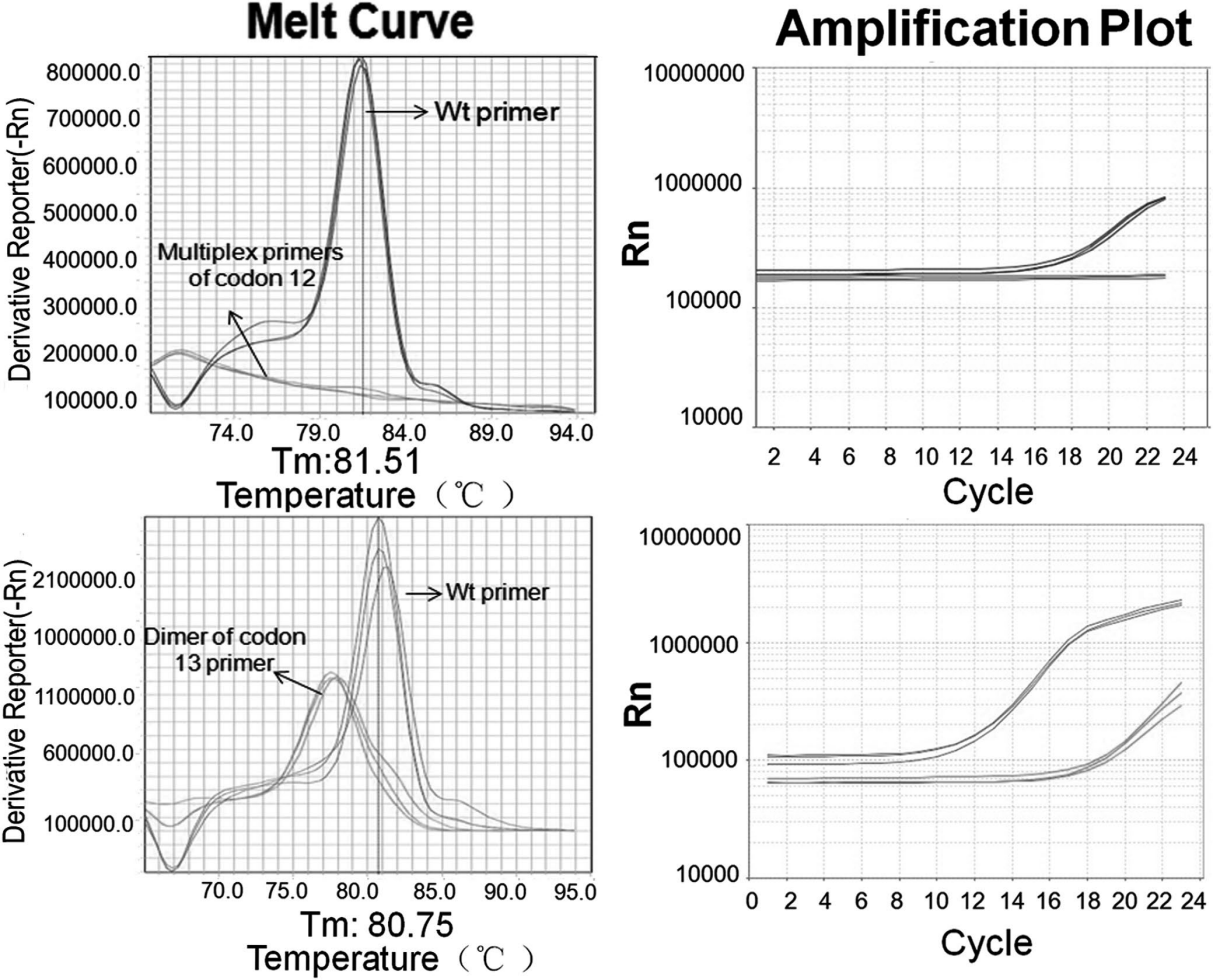
The current assay would not obtain false‐positive signals of *KRAS* gene mutations in pooled DNA sample of health control. As the top melt curve and amplification plot shows, the pooled DNA sample could be amplified by the wild‐type primers but not be amplified by the codon 12 mutation primers of *KRAS*. As the bottom melt curve and amplification plot shows, the DNA pool sample could be amplified by the *KRAS* wild‐type primers but could not be amplified by the codon 13 mutation primers of *KRAS*

This assay was further tested for its application in clinical samples. The efficient extension of *KRAS* mutation primers would not be observed in healthy human genomic DNA samples. As shown in Figure 3, we used one pooled DNA sample of 200 normal individuals for evaluating the specificity of our method. Mutation‐specific primers failed to amplify products in these samples, while products were amplified by wild‐type‐specific detection primers. Codon 13 primers of *KRAS* could amplify a primer dimer, and we could distinguish the objective fragments from the dimer according to the Tm value. To further confirm this method, the PCR products of the *KRAS* gene were sent for sequencing and there were no point mutations in codon 12 and codon 13 of *KRAS* gene.

### 3.4 Application of the current assay in *KRAS* hotspot mutation detection with cancer patients

**Figure 4:**
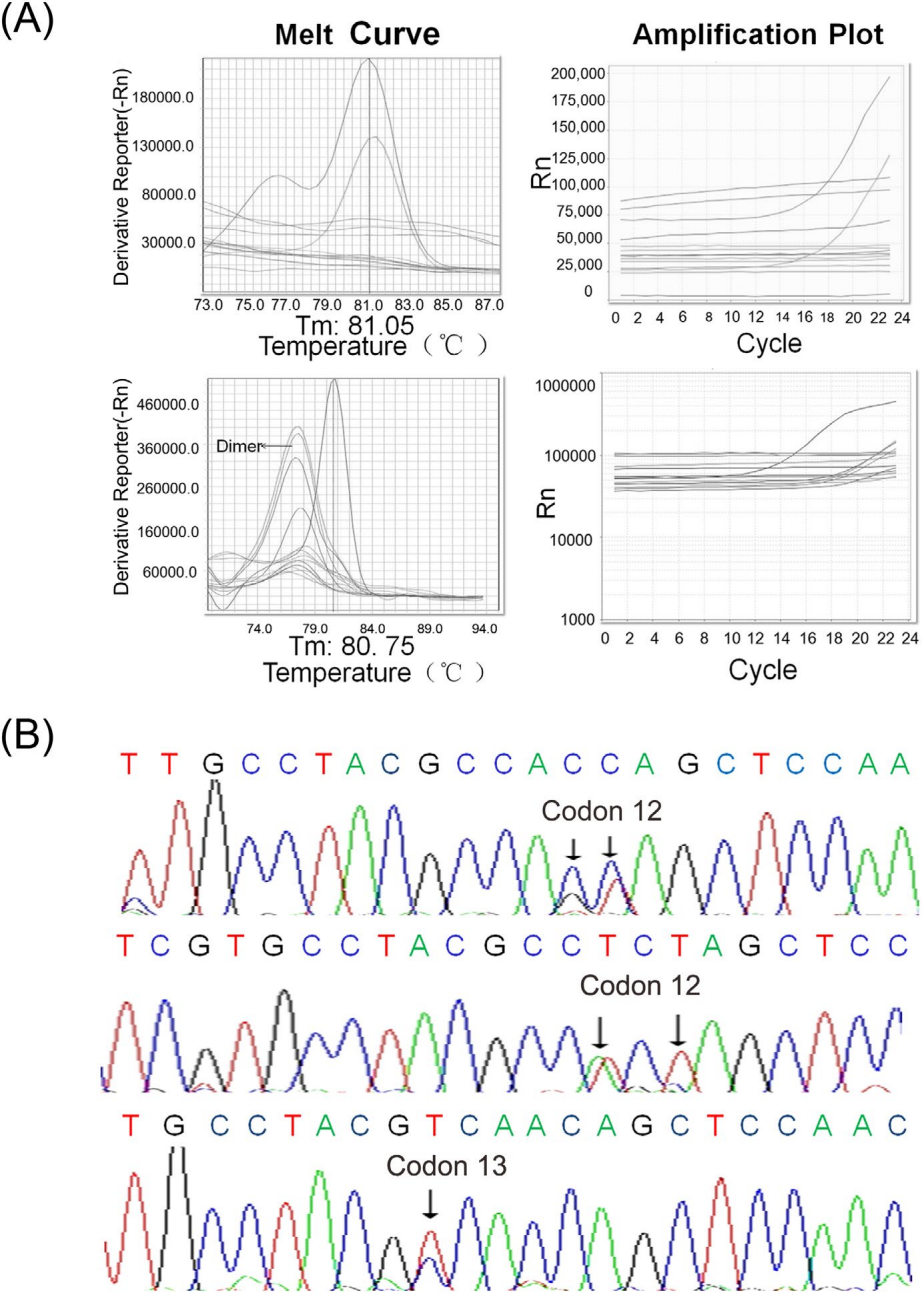
The feasibility was tested for 7 hotspot mutations detecting of *KRAS* gene in cancer patients ctDNA samples. A, As the top melt curve and amplification plot shows, two of fourteen samples could be detected the codon 12 mutations of *KRAS* gene; as the bottom melt curve and amplification plot shows, one of fourteen samples could be detected the codon 13 mutation of *KRAS* gene. B, The sequencing of *KRAS* gene contained codon 12 and codon 13 of the three samples

CtDNA samples derived from fourteen tumor patients were tested with the newly developed *KRAS* hotspot mutation assay, two samples were detected carrying at least one type mutations of codon 12 and one sample was detected carrying codon 13 mutation according to the amplification curve and melt curve. The Sanger sequencing confirmed the *KRAS* gene mutations in the three cancer patients (Figure 4B): codon 12 mutations (GGT>AGT, GGT>GCT), codon 12 mutations (GGT>AGT, GGT>AGA), and mutation in codon 13 (GGC>GAC). Two of the three cancer patients carrying the *KRAS* gene mutations suffered from colorectal cancer surgery and adjuvant chemotherapy, and another case is lung cancer without chemotherapy. The other 11 samples could not be detected by our assay and had been confirmed by the sequencing. It indicates that the assay system we developed is highly sensitive and specific in mutation detection with clinical samples, especially for ctDNA samples.

## 4 DISCUSSION

The *KRAS* mutations are extremely rare in normal individuals, while they are presented in about 30% of cancer patients. High specificity and sensitivity of assays are crucial to avoid or minimize false‐positive detection in normal DNA samples and false‐negative results in patient samples. Sequencing is the most common technique to screen *KRAS* mutations, but it requires at least 10%‐30% abundance of mutated templates.^10^, ^28^ Screening the *KRAS* mutations by HRM had a sensitivity of 5%‐6%.^10^, ^28^ Hillary et al were able to detect *KRAS* mutations by allele‐specific hybridization‐induced aggregation (HIA) of oligonucleotide probe‐conjugated microbeads with a sensitivity of 25%.^29^ Our multiplex real‐time PCR method can detect *KRAS* mutation as few as 100 copies in at least 10^5^ wild‐type counterparts. Currently, ctDNA concentrations vary from 0 to 1000 ng/mL blood, with the median concentration of cfDNA 790 ng/mL.^30^, ^31^ In general, 1000 ng cfDNA includes approximately 150 000 copies of the genome^31^; accordingly to this, it is difficult to cause false‐positive amplification in one PCR system by our assay.

In the present study, efficient primer extension was exclusively observed when the mutation‐specific primers matched the mutation templates. One pooled DNA sample 200 normal individuals was used to test the newly developed assay, only wild‐type primers but not mutation‐specific primers was able to amplify the pooled DNA sample. For the 14 ctDNA samples of cancer patients, 3 of them generated PCR products when mutation‐specific primers were used, and the presence of point mutations of *KRAS* in the 3 samples was further confirmed by DNA sequencing. It indicated the accuracy of the newly developed multiplex PCR system in genetic diagnostics. The high‐fidelity DNA polymerase ensures the high sensitivity and specificity of the assay. This assay and similar assays have great potential to be widely applied in genetic diagnostics in clinic, especially for somatic mutations. In addition, multiplex PCR is time‐efficient and less financial consuming.

## 5 CONCLUSION

The present study has successfully established a non‐invasive diagnostic method for *KRAS* mutation detection in circulating DNA of cancer patients. The allele‐specific real‐time PCR is mediated by mutation‐sensitive “molecular switch” composed of high‐fidelity DNA polymerase and phosphorylated‐modified primers. We have demonstrated the success of using the assay in micro‐mutation detection with ctDNA. This technology has a great potential in screening *KRAS* gene mutations thanks to its high specificity, sensitivity, low‐cost, multiplex detection, and easy data interpretation.

## CONFLICT OF INTEREST

No competing financial interests exist.

## Supporting information

Supplementary Material

Data S1
