# Supplementary material for: An enzymatic on/off switch‐mediated assay for KRAS hotspot point mutation detection of circulating tumor DNA
Source: J Clin Lab Anal. 2020 Mar 24;34(8):e23305. doi: 10.1002/jcla.23305 (PMC7439329; doi:10.1002/jcla.23305)
Supplement: Supplementary file 1 — Supplementary Material [file JCLA-34-e23305-s001.docx]

**Supplementary table 1. Design sixteen primers for seven mutation genetype templates**

| T1KRASmut: | 5’–GAACAGCAGTCTGGCTATTTA–3’ |
| --- | --- |
| T2KRASmut: | 5’–TCTGTATCAAAGAATGGTCC–3’ |
| T3KRASmut: | 5’–TGCCTACGCCAACAGCTCCAACTA–3’ |
| T4KRASmut: | 5’–TAGTTGGAGCTGTTGGCGTAGGCA–3’ |
| T5KRASmut: | 5’–TGCCTACGCCATCAGCTCCAACTA–3’ |
| T6KRASmut: | 5’–TAGTTGGAGCTGATGGCGTAGGCA–3’ |
| T7KRASmut: | 5’–GGCACTCTTGCCTACGTCACCAGCTCCAACTA–3’ |
| T8KRASmut: | 5’–TAGTTGGAGCTGGTGACGTAGGCAAGAGTGCC–3’ |
| T9KRASmut: | 5’–TGCCTACGCCAGCAGCTCCAACTA–3’ |
| T10KRASmut: | 5’–TAGTTGGAGCTGCTGGCGTAGGCA–3’ |
| T11KRASmut: | 5’–TGCCTACGCCACTAGCTCCAACTA–3’ |
| T12KRASmut: | 5’–TAGTTGGAGCTAGTGGCGTAGGCA–3’ |
| T13KRASmut: | 5’–TGCCTACGCCACAAGCTCCAACTA–3’ |
| T14KRASmut: | 5’–TAGTTGGAGCTTGTGGCGTAGGCA–3’ |
| T15KRASmut: | 5’–TGCCTACGCCACGAGCTCCAACTA–3’ |
| T16KRASmut: | 5’–TAGTTGGAGCTCGTGGCGTAGGCA–3’ |
| FC | 5’-TGAATATAAACTTGTGGTAG–3’ |
| RC | 5’–TCTGTATCAAAGAATGGTCCTGC-3’ |

Note: T1KRASmut and T3KRASmut, T5KRASmut, T7KRASmut, T9KRASmut, T11KRASmut, T13KRASmut, T15KRASmut were used to amplify the upstream fragments, and the common primer T2KRASmut and T4KRASmut, T6KRASmut, T8KRASmut, T10KRASmut, T12KRASmut, T14KRASmut or T16KRASmut were used to amplify the downstream fragments of the seven mutations of the KRAS gene including G12D，G12V，G12S，G12C，G12R, G12A and G13D.


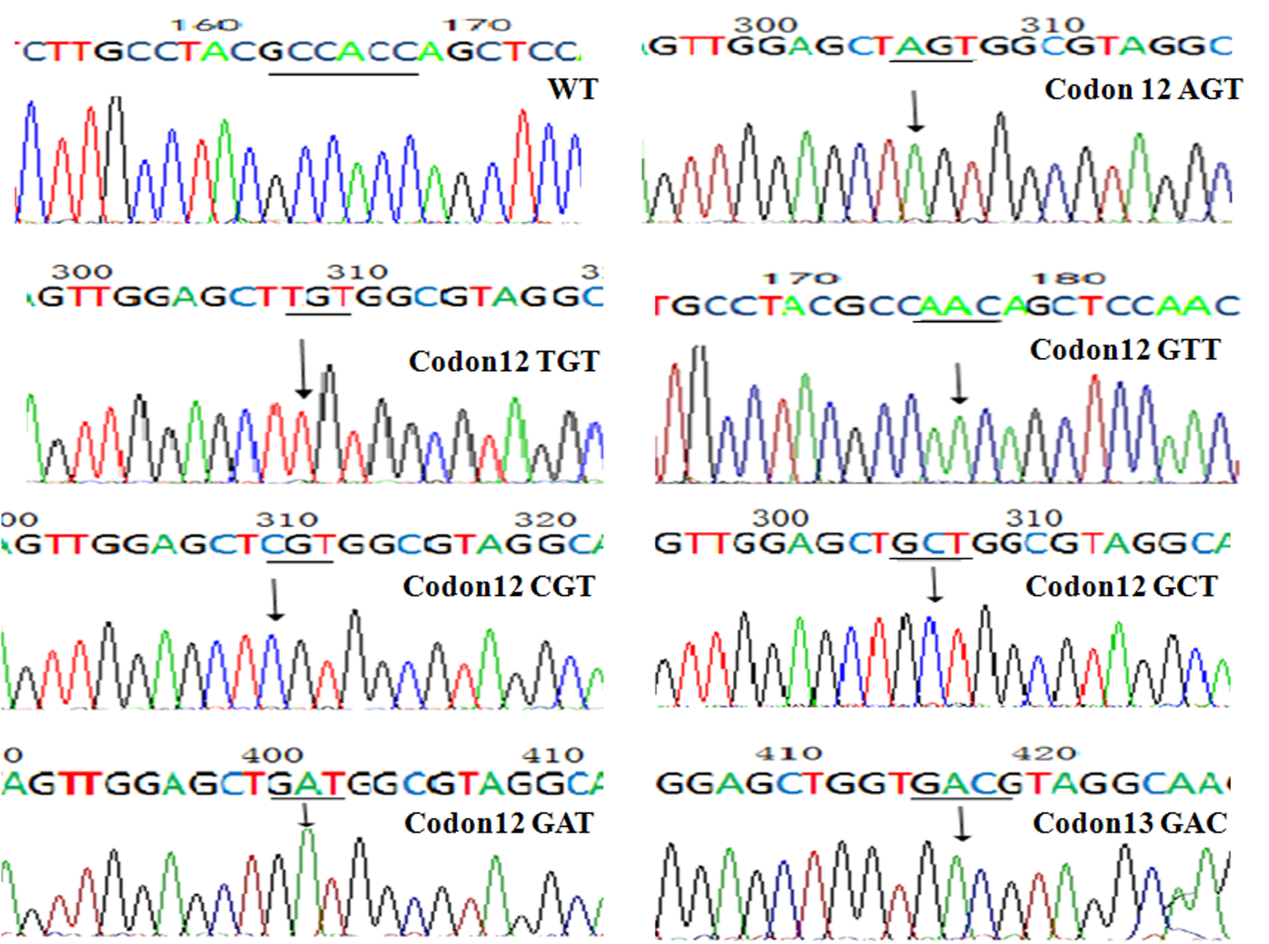


Supplementary figure 1 The seven mutations plasimd templates of *KRAS* gene including G12D，G12V，G12S，G12C，G12R, G12A，and G13D.
