# Supplementary material for: An enzymatic on/off switch‐mediated assay for KRAS hotspot point mutation detection of circulating tumor DNA
Source: J Clin Lab Anal. 2020 Mar 24;34(8):e23305. doi: 10.1002/jcla.23305 (PMC7439329; doi:10.1002/jcla.23305)
Supplement: Supplementary file 2 — Data S1 [file JCLA-34-e23305-s002.docx]

|  |  |  |  |  |
| --- | --- | --- | --- | --- |
| kras_event = 1 mutation type，2 wild type. | | | |  |
|  |  |  |  |  |
| id | fustat | futime | kras_event | KRAS |
| TCGA-3L-AA1B | 0 | 154 | 1 | 1386.3636 |
| TCGA-4N-A93T | 0 | 8 | 1 | 1250.1209 |
| TCGA-4T-AA8H | 0 | 160 | 1 | 1689.6363 |
| TCGA-5M-AAT4 | 1 | 49 | 1 | 885.5422 |
| TCGA-5M-AAT6 | 1 | 290 | 1 | 1797.4299 |
| TCGA-5M-AATE | 0 | 1200 | 2 | 739.3398 |
| TCGA-A6-2671 | 0 | 648 | 1 | 1898.9518 |
| TCGA-A6-2675 | 0 | 515 | 1 | 2001.1154 |
| TCGA-A6-2675 | 0 | 515 | 1 | 1193.7964 |
| TCGA-A6-2678 | 0 | 437 | 1 | 1741.9292 |
| TCGA-A6-2679 | 0 | 595 | 1 | 1663.5329 |
| TCGA-A6-2680 | 0 | 522 | 1 | 1386.2096 |
| TCGA-A6-2682 | 1 | 424 | 2 | 1801.8782 |
| TCGA-A6-2682 | 1 | 424 | 2 | 1614.2067 |
| TCGA-A6-2682 | 1 | 424 | 2 | 1801.8782 |
| TCGA-A6-2682 | 1 | 424 | 2 | 1614.2067 |
| TCGA-A6-2683 | 0 | 472 | 2 | 1882.5273 |
| TCGA-A6-2684 | 0 | 387 | 1 | 1571.6919 |
| TCGA-A6-2684 | 0 | 387 | 1 | 1150.9017 |
| TCGA-A6-2685 | 0 | 464 | 1 | 2091.2473 |
| TCGA-A6-2685 | 0 | 464 | 1 | 1656.8154 |
| TCGA-A6-2686 | 0 | 488 | 1 | 1928.7756 |
| TCGA-A6-2686 | 0 | 488 | 1 | 974.1589 |
| TCGA-A6-4105 | 1 | 442 | 1 | 1441.4969 |
| TCGA-A6-4105 | 1 | 442 | 1 | 1441.4969 |
| TCGA-A6-5656 | 0 | 462 | 1 | 935.8407 |
| TCGA-A6-5657 | 0 | 367 | 1 | 926.6226 |
| TCGA-A6-5659 | 0 | 286 | 1 | 2594.6554 |
| TCGA-A6-5659 | 0 | 286 | 1 | 1092.0897 |
| TCGA-A6-5660 | 0 | 342 | 1 | 1069.5876 |
| TCGA-A6-5661 | 0 | 306 | 1 | 1769.0418 |
| TCGA-A6-5662 | 0 | 297 | 2 | 1779.8216 |
| TCGA-A6-5662 | 0 | 297 | 2 | 1450.6494 |
| TCGA-A6-5664 | 0 | 362 | 1 | 1624.7519 |
| TCGA-A6-5665 | 0 | 286 | 1 | 2540.5539 |
| TCGA-A6-5665 | 0 | 286 | 1 | 1233.2475 |
| TCGA-A6-5666 | 0 | 244 | 1 | 1600.6113 |
| TCGA-A6-5667 | 0 | 320 | 1 | 1805.9152 |
| TCGA-A6-5667 | 0 | 320 | 1 | 1011.1288 |
| TCGA-A6-6137 | 0 | 195 | 1 | 1344.4098 |
| TCGA-A6-6138 | 0 | 182 | 1 | 1165.7609 |
| TCGA-A6-6140 | 0 | 125 | 1 | 1043.535 |
| TCGA-A6-6141 | 0 | 130 | 1 | 1419.6525 |
| TCGA-A6-6142 | 0 | 134 | 2 | 1014.751 |
| TCGA-A6-6648 | 0 | 253 | 2 | 1722.9987 |
| TCGA-A6-6649 | 0 | 147 | 1 | 1181.2166 |
| TCGA-A6-6649 | 0 | 147 | 1 | 1181.2166 |
| TCGA-A6-6650 | 0 | 173 | 1 | 1420.6696 |
| TCGA-A6-6651 | 0 | 249 | 1 | 932.6772 |
| TCGA-A6-6652 | 0 | 135 | 1 | 710.8381 |
| TCGA-A6-6653 | 0 | 91 | 1 | 1060.6787 |
| TCGA-A6-6654 | 0 | 178 | 1 | 1010.7823 |
| TCGA-A6-6780 | 0 | 87 | 1 | 1544.7355 |
| TCGA-A6-6781 | 0 | 158 | 1 | 981.8269 |
| TCGA-A6-6782 | 0 | 164 | 1 | 1174.1623 |
| TCGA-A6-A565 | 1 | 494 | 2 | 1995.9335 |
| TCGA-A6-A566 | 1 | 758 | 2 | 1828.8558 |
| TCGA-A6-A567 | 1 | 1881 | 2 | 1104.7324 |
| TCGA-A6-A56B | 0 | 1595 | 2 | 794.2925 |
| TCGA-A6-A5ZU | 0 | 117 | 1 | 928.1177 |
| TCGA-AA-3489 | 0 | 31 | 1 | 1810.0907 |
| TCGA-AA-3489 | 0 | 31 | 1 | 1209.4082 |
| TCGA-AA-3492 | 0 | 0 | 1 | 1142.9423 |
| TCGA-AA-3495 | 0 | 31 | 1 | 974.5239 |
| TCGA-AA-3496 | 0 | 31 | 1 | 1621.2575 |
| TCGA-AA-3496 | 0 | 31 | 1 | 1134.6948 |
| TCGA-AA-3496 | 0 | 31 | 1 | 1621.2575 |
| TCGA-AA-3496 | 0 | 31 | 1 | 1134.6948 |
| TCGA-AA-3502 | 0 | 31 | 1 | 1231.8841 |
| TCGA-AA-3506 | 0 | 0 | 1 | 854.0285 |
| TCGA-AA-3506 | 0 | 0 | 1 | 854.0285 |
| TCGA-AA-3509 | 0 | 0 | 1 | 951.4542 |
| TCGA-AA-3511 | 0 | 0 | 1 | 1975.3336 |
| TCGA-AA-3511 | 0 | 0 | 1 | 1097.4282 |
| TCGA-AA-3514 | 0 | 0 | 1 | 2158.5586 |
| TCGA-AA-3516 | 0 | 0 | 1 | 1878.5444 |
| TCGA-AA-3516 | 0 | 0 | 1 | 1878.5444 |
| TCGA-AA-3517 | 0 | 0 | 1 | 1898.8353 |
| TCGA-AA-3518 | 0 | 31 | 1 | 1793.9579 |
| TCGA-AA-3520 | 0 | 0 | 1 | 2142.8571 |
| TCGA-AA-3520 | 0 | 0 | 1 | 2142.8571 |
| TCGA-AA-3522 | 0 | 0 | 1 | 2092.031 |
| TCGA-AA-3522 | 0 | 0 | 1 | 2092.031 |
| TCGA-AA-3525 | 0 | 0 | 1 | 1982.8234 |
| TCGA-AA-3525 | 0 | 0 | 1 | 1982.8234 |
| TCGA-AA-3526 | 0 | 30 | 1 | 953.2753 |
| TCGA-AA-3527 | 0 | 0 | 1 | 2142.0077 |
| TCGA-AA-3527 | 0 | 0 | 1 | 2142.0077 |
| TCGA-AA-3531 | 0 | 0 | 1 | 2650.3502 |
| TCGA-AA-3531 | 0 | 0 | 1 | 2650.3502 |
| TCGA-AA-3534 | 0 | 0 | 1 | 1784.7913 |
| TCGA-AA-3655 | 0 | 0 | 1 | 1755.122 |
| TCGA-AA-3655 | 0 | 0 | 1 | 1534.0751 |
| TCGA-AA-3660 | 0 | 31 | 1 | 2276.206 |
| TCGA-AA-3660 | 0 | 31 | 1 | 1635.2657 |
| TCGA-AA-3662 | 0 | 0 | 1 | 1792.4503 |
| TCGA-AA-3662 | 0 | 0 | 1 | 1056.7089 |
| TCGA-AA-3662 | 0 | 0 | 1 | 1792.4503 |
| TCGA-AA-3662 | 0 | 0 | 1 | 1056.7089 |
| TCGA-AA-3663 | 0 | 31 | 1 | 1539.0124 |
| TCGA-AA-3663 | 0 | 31 | 1 | 890.6593 |
| TCGA-AA-3675 | 0 | 31 | 1 | 907.4819 |
| TCGA-AA-3685 | 0 | 31 | 1 | 883.9458 |
| TCGA-AA-3697 | 0 | 30 | 1 | 1885.2814 |
| TCGA-AA-3697 | 0 | 30 | 1 | 1064.3963 |
| TCGA-AA-3697 | 0 | 30 | 1 | 1885.2814 |
| TCGA-AA-3697 | 0 | 30 | 1 | 1064.3963 |
| TCGA-AA-3712 | 0 | 0 | 1 | 1853.9631 |
| TCGA-AA-3712 | 0 | 0 | 1 | 1368.6097 |
| TCGA-AA-3713 | 0 | 30 | 1 | 1634.6966 |
| TCGA-AA-3713 | 0 | 30 | 1 | 1452.7695 |
| TCGA-AA-3713 | 0 | 30 | 1 | 1634.6966 |
| TCGA-AA-3713 | 0 | 30 | 1 | 1452.7695 |
| TCGA-AA-A01P | 0 | 0 | 1 | 991.7995 |
| TCGA-AA-A01X | 0 | 30 | 1 | 1039.2523 |
| TCGA-AA-A01Z | 0 | 0 | 1 | 1220.1717 |
| TCGA-AA-A02K | 0 | 0 | 1 | 1185.726 |
| TCGA-AA-A02Y | 0 | 31 | 1 | 1169.4552 |
| TCGA-AD-5900 | 0 | 2 | 2 | 1046.6805 |
| TCGA-AD-6548 | 0 | 3 | 2 | 1134.5113 |
| TCGA-AD-6548 | 0 | 3 | 2 | 1134.5113 |
| TCGA-AD-6888 | 0 | 155 | 1 | 683.6242 |
| TCGA-AD-6888 | 0 | 155 | 1 | 683.6242 |
| TCGA-AD-6889 | 0 | 1720 | 1 | 971.8382 |
| TCGA-AD-6890 | 0 | 24 | 1 | 910.7565 |
| TCGA-AD-6895 | 0 | 22 | 1 | 1466.9055 |
| TCGA-AD-6895 | 0 | 22 | 1 | 1466.9055 |
| TCGA-AD-6899 | 0 | 48 | 1 | 842.7606 |
| TCGA-AD-6901 | 0 | 31 | 1 | 774.0052 |
| TCGA-AD-6963 | 0 | 3 | 1 | 1312.3903 |
| TCGA-AD-6964 | 0 | 23 | 1 | 1354.1794 |
| TCGA-AD-6964 | 0 | 23 | 1 | 1354.1794 |
| TCGA-AD-6965 | 0 | 6 | 1 | 1157.5413 |
| TCGA-AD-A5EJ | 0 | 0 | 2 | 1628.655 |
| TCGA-AD-A5EK | 0 | 13 | 1 | 1017.6245 |
| TCGA-AM-5820 | 0 | 14 | 1 | 1676.8267 |
| TCGA-AM-5821 | 0 | 28 | 1 | 1530.7601 |
| TCGA-AU-3779 | 0 | 441 | 1 | 1329.6846 |
| TCGA-AU-6004 | 0 | 203 | 1 | 1154.9142 |
| TCGA-AY-5543 | 0 | 230 | 1 | 1569.4006 |
| TCGA-AY-6196 | 0 | 6 | 1 | 1123.0492 |
| TCGA-AY-6197 | 0 | 113 | 1 | 1146.7702 |
| TCGA-AY-6386 | 0 | 169 | 1 | 1821.2179 |
| TCGA-AY-A54L | 0 | 158 | 2 | 1327.3961 |
| TCGA-AY-A69D | 0 | 168 | 1 | 1793.1279 |
| TCGA-AY-A71X | 0 | 235 | 1 | 1243.2744 |
| TCGA-AY-A8YK | 0 | 209 | 1 | 1418.2388 |
| TCGA-AZ-4313 | 0 | 957 | 2 | 1332.8605 |
| TCGA-AZ-4313 | 0 | 957 | 2 | 1332.8605 |
| TCGA-AZ-4315 | 0 | 1334 | 2 | 1005.8557 |
| TCGA-AZ-4323 | 1 | 43 | 2 | 1758.8759 |
| TCGA-AZ-4614 | 1 | 172 | 2 | 710.7843 |
| TCGA-AZ-4615 | 0 | 506 | 2 | 918.6197 |
| TCGA-AZ-4616 | 1 | 156 | 2 | 1174.8879 |
| TCGA-AZ-4682 | 1 | 680 | 2 | 931.2993 |
| TCGA-AZ-4684 | 0 | 1705 | 2 | 767.8974 |
| TCGA-AZ-5403 | 1 | 1910 | 2 | 1420.6386 |
| TCGA-AZ-5407 | 0 | 1395 | 2 | 985.963 |
| TCGA-AZ-6598 | 1 | 1503 | 2 | 1540.5242 |
| TCGA-AZ-6598 | 1 | 1503 | 2 | 1471.3823 |
| TCGA-AZ-6599 | 1 | 206 | 2 | 1896.4578 |
| TCGA-AZ-6599 | 1 | 206 | 2 | 1292.0647 |
| TCGA-AZ-6600 | 1 | 368 | 2 | 2525.2554 |
| TCGA-AZ-6600 | 1 | 368 | 2 | 1396.2664 |
| TCGA-AZ-6601 | 1 | 3042 | 2 | 1993.8776 |
| TCGA-AZ-6601 | 1 | 3042 | 2 | 1207.173 |
| TCGA-AZ-6601 | 1 | 3042 | 2 | 1993.8776 |
| TCGA-AZ-6601 | 1 | 3042 | 2 | 1207.173 |
| TCGA-AZ-6603 | 1 | 899 | 2 | 1222.1053 |
| TCGA-AZ-6603 | 1 | 899 | 2 | 1049.4561 |
| TCGA-AZ-6605 | 1 | 159 | 2 | 1344.7077 |
| TCGA-AZ-6605 | 1 | 159 | 2 | 1177.1654 |
| TCGA-AZ-6606 | 1 | 357 | 2 | 1917.455 |
| TCGA-AZ-6607 | 1 | 97 | 2 | 1947.983 |
| TCGA-AZ-6608 | 1 | 59 | 2 | 936.7347 |
| TCGA-AZ-6608 | 1 | 59 | 2 | 936.7347 |
| TCGA-CA-5254 | 0 | 5 | 2 | 1965.3376 |
| TCGA-CA-5255 | 0 | 2 | 2 | 1902.3307 |
| TCGA-CA-5256 | 0 | 0 | 1 | 828.4243 |
| TCGA-CA-5796 | 0 | 12 | 2 | 1965.4952 |
| TCGA-CA-5797 | 0 | 8 | 2 | 871.9762 |
| TCGA-CA-6715 | 0 | 2 | 2 | 785.6615 |
| TCGA-CA-6716 | 0 | 8 | 2 | 1238.5701 |
| TCGA-CA-6717 | 0 | 10 | 2 | 1475.4615 |
| TCGA-CA-6718 | 0 | 3 | 2 | 1229.6238 |
| TCGA-CA-6719 | 0 | 41 | 2 | 1000.4636 |
| TCGA-CK-4947 | 0 | 534 | 1 | 1500.8453 |
| TCGA-CK-4948 | 0 | 3116 | 1 | 1253.6184 |
| TCGA-CK-4950 | 0 | 1227 | 1 | 1523.9537 |
| TCGA-CK-4951 | 0 | 1492 | 1 | 1110.5974 |
| TCGA-CK-4952 | 0 | 475 | 1 | 1124.4372 |
| TCGA-CK-5912 | 0 | 1466 | 1 | 1150.6986 |
| TCGA-CK-5912 | 0 | 1466 | 1 | 1150.6986 |
| TCGA-CK-5913 | 0 | 853 | 1 | 1612.5368 |
| TCGA-CK-5914 | 0 | 669 | 1 | 1247.8736 |
| TCGA-CK-5915 | 0 | 0 | 1 | 1153.1972 |
| TCGA-CK-5916 | 1 | 643 | 1 | 1885.4889 |
| TCGA-CK-6746 | 0 | 0 | 1 | 1409.2153 |
| TCGA-CK-6747 | 0 | 89 | 1 | 1176.9633 |
| TCGA-CK-6748 | 0 | 61 | 2 | 1717.1307 |
| TCGA-CK-6751 | 0 | 518 | 1 | 1315.1101 |
| TCGA-CM-4743 | 0 | 184 | 1 | 1366.7497 |
| TCGA-CM-4744 | 0 | 214 | 1 | 2615.0056 |
| TCGA-CM-4747 | 0 | 244 | 1 | 1610.8706 |
| TCGA-CM-4751 | 0 | 607 | 1 | 1384.8263 |
| TCGA-CM-5344 | 0 | 304 | 1 | 1547.0349 |
| TCGA-CM-5348 | 0 | 393 | 1 | 1099.0764 |
| TCGA-CM-5349 | 0 | 335 | 1 | 1732.052 |
| TCGA-CM-5860 | 0 | 304 | 1 | 1417.8929 |
| TCGA-CM-5861 | 0 | 335 | 1 | 1339.9293 |
| TCGA-CM-5862 | 1 | 153 | 2 | 941.1765 |
| TCGA-CM-5863 | 0 | 304 | 1 | 1434.9603 |
| TCGA-CM-5864 | 0 | 61 | 1 | 818.1199 |
| TCGA-CM-5868 | 0 | 304 | 2 | 969.5341 |
| TCGA-CM-6161 | 0 | 212 | 1 | 812.848 |
| TCGA-CM-6162 | 0 | 212 | 1 | 1843.4116 |
| TCGA-CM-6163 | 0 | 90 | 1 | 1247.8541 |
| TCGA-CM-6164 | 0 | 487 | 1 | 1040.4153 |
| TCGA-CM-6165 | 0 | 212 | 1 | 1295.421 |
| TCGA-CM-6166 | 0 | 424 | 1 | 1550.2994 |
| TCGA-CM-6167 | 0 | 122 | 1 | 1754.637 |
| TCGA-CM-6168 | 0 | 181 | 1 | 1401.7274 |
| TCGA-CM-6169 | 0 | 243 | 1 | 1288.8425 |
| TCGA-CM-6170 | 0 | 242 | 1 | 1342.6195 |
| TCGA-CM-6171 | 0 | 182 | 1 | 1984.4787 |
| TCGA-CM-6172 | 0 | 212 | 1 | 1213.6337 |
| TCGA-CM-6674 | 0 | 150 | 1 | 993.7923 |
| TCGA-CM-6675 | 0 | 153 | 1 | 993.2357 |
| TCGA-CM-6676 | 0 | 153 | 1 | 1334.7639 |
| TCGA-CM-6677 | 0 | 153 | 1 | 1050.1016 |
| TCGA-CM-6678 | 0 | 122 | 2 | 1359.0078 |
| TCGA-CM-6679 | 0 | 122 | 1 | 720.2052 |
| TCGA-CM-6680 | 0 | 91 | 1 | 1296.7211 |
| TCGA-D5-5537 | 0 | 456 | 1 | 1278.7331 |
| TCGA-D5-5538 | 0 | 204 | 1 | 1798.6411 |
| TCGA-D5-5539 | 0 | 30 | 1 | 1328.0094 |
| TCGA-D5-5540 | 0 | 198 | 1 | 1514.1626 |
| TCGA-D5-5541 | 0 | 202 | 1 | 850.7871 |
| TCGA-D5-6529 | 0 | 260 | 1 | 1725.7248 |
| TCGA-D5-6530 | 0 | 271 | 1 | 1542.5477 |
| TCGA-D5-6531 | 0 | 17 | 1 | 1321.2508 |
| TCGA-D5-6532 | 0 | 107 | 1 | 1061.9508 |
| TCGA-D5-6533 | 0 | 40 | 1 | 1123.119 |
| TCGA-D5-6534 | 0 | 241 | 1 | 1039.6354 |
| TCGA-D5-6535 | 0 | 10 | 1 | 2062.599 |
| TCGA-D5-6536 | 0 | 287 | 1 | 1092.9812 |
| TCGA-D5-6537 | 0 | 137 | 1 | 1368.9368 |
| TCGA-D5-6538 | 0 | 246 | 1 | 762.7385 |
| TCGA-D5-6539 | 0 | 145 | 1 | 2066.3252 |
| TCGA-D5-6540 | 0 | 186 | 1 | 1185.8801 |
| TCGA-D5-6541 | 0 | 108 | 1 | 908.656 |
| TCGA-D5-6898 | 0 | 45 | 1 | 1121.2737 |
| TCGA-D5-6920 | 0 | 112 | 1 | 1364.1732 |
| TCGA-D5-6922 | 0 | 134 | 1 | 869.4087 |
| TCGA-D5-6923 | 0 | 43 | 1 | 995.256 |
| TCGA-D5-6924 | 0 | 89 | 1 | 965.7681 |
| TCGA-D5-6926 | 0 | 95 | 1 | 713.677 |
| TCGA-D5-6927 | 0 | 67 | 1 | 915.5267 |
| TCGA-D5-6928 | 0 | 20 | 1 | 1057.9216 |
| TCGA-D5-6929 | 0 | 146 | 1 | 1183.334 |
| TCGA-D5-6930 | 0 | 46 | 1 | 857.9491 |
| TCGA-D5-6931 | 0 | 43 | 1 | 1884.0037 |
| TCGA-D5-6932 | 0 | 19 | 1 | 683.8841 |
| TCGA-D5-7000 | 0 | 41 | 1 | 1826.5216 |
| TCGA-DM-A0X9 | 0 | 3429 | 2 | 1296.8198 |
| TCGA-DM-A0XD | 1 | 743 | 2 | 1372.2423 |
| TCGA-DM-A0XF | 1 | 1162 | 2 | 913.3994 |
| TCGA-DM-A1D0 | 0 | 3625 | 2 | 1272.8181 |
| TCGA-DM-A1D4 | 1 | 2821 | 1 | 1421.9323 |
| TCGA-DM-A1D6 | 1 | 570 | 2 | 1703.5155 |
| TCGA-DM-A1D7 | 1 | 0 | 2 | 783.5168 |
| TCGA-DM-A1D8 | 1 | 383 | 2 | 621.077 |
| TCGA-DM-A1D9 | 0 | 3920 | 1 | 1578.1457 |
| TCGA-DM-A1DA | 1 | 228 | 2 | 668.1514 |
| TCGA-DM-A1DB | 1 | 1348 | 2 | 1233.8351 |
| TCGA-DM-A1HA | 0 | 2600 | 2 | 1379.9162 |
| TCGA-DM-A1HB | 0 | 3786 | 2 | 811.2029 |
| TCGA-DM-A280 | 1 | 236 | 2 | 1523.1882 |
| TCGA-DM-A282 | 0 | 4122 | 2 | 924.029 |
| TCGA-DM-A285 | 1 | 179 | 2 | 1089.4502 |
| TCGA-DM-A285 | 1 | 179 | 2 | 1089.4502 |
| TCGA-DM-A288 | 1 | 427 | 2 | 1272.2946 |
| TCGA-DM-A28A | 1 | 805 | 2 | 991.9797 |
| TCGA-DM-A28C | 1 | 2475 | 1 | 835.9439 |
| TCGA-DM-A28E | 0 | 3530 | 2 | 1220.476 |
| TCGA-DM-A28F | 1 | 1094 | 2 | 869.0652 |
| TCGA-DM-A28G | 1 | 1849 | 2 | 1111.0092 |
| TCGA-DM-A28H | 0 | 3423 | 2 | 1211.8577 |
| TCGA-DM-A28K | 0 | 2835 | 1 | 1299.7925 |
| TCGA-DM-A28M | 0 | 2775 | 2 | 1499.2727 |
| TCGA-F4-6459 | 0 | 0 | 1 | 1982.4861 |
| TCGA-F4-6460 | 0 | 0 | 1 | 1017.7268 |
| TCGA-F4-6461 | 0 | 0 | 1 | 1106.9638 |
| TCGA-F4-6463 | 0 | 0 | 1 | 1518.5 |
| TCGA-F4-6569 | 0 | 0 | 1 | 1152.194 |
| TCGA-F4-6570 | 0 | 0 | 1 | 762.1039 |
| TCGA-F4-6703 | 0 | 56 | 2 | 1507.4342 |
| TCGA-F4-6704 | 0 | 47 | 1 | 2237.1062 |
| TCGA-F4-6704 | 0 | 47 | 1 | 938.9633 |
| TCGA-F4-6805 | 0 | 16 | 1 | 1698.7224 |
| TCGA-F4-6806 | 0 | 20 | 1 | 1482.2505 |
| TCGA-F4-6807 | 0 | 16 | 1 | 1305.0672 |
| TCGA-F4-6808 | 0 | 14 | 1 | 660.1539 |
| TCGA-F4-6809 | 0 | 39 | 1 | 1300.8475 |
| TCGA-F4-6854 | 0 | 16 | 1 | 879.5066 |
| TCGA-F4-6855 | 0 | 42 | 2 | 910.4893 |
| TCGA-F4-6856 | 0 | 20 | 1 | 1233.5304 |
| TCGA-G4-6293 | 0 | 3203 | 1 | 1376.9883 |
| TCGA-G4-6294 | 1 | 858 | 1 | 1161.8584 |
| TCGA-G4-6294 | 1 | 858 | 1 | 1161.8584 |
| TCGA-G4-6295 | 0 | 254 | 1 | 990.8881 |
| TCGA-G4-6297 | 0 | 2289 | 1 | 2049.8562 |
| TCGA-G4-6298 | 1 | 0 | 1 | 704.343 |
| TCGA-G4-6298 | 1 | 0 | 1 | 704.343 |
| TCGA-G4-6299 | 0 | 2077 | 1 | 931.3012 |
| TCGA-G4-6302 | 0 | 0 | 1 | 1120.603 |
| TCGA-G4-6303 | 0 | 1882 | 2 | 1124.6666 |
| TCGA-G4-6304 | 0 | 1631 | 1 | 634.9345 |
| TCGA-G4-6306 | 0 | 1359 | 1 | 971.8508 |
| TCGA-G4-6307 | 0 | 1485 | 1 | 691.384 |
| TCGA-G4-6309 | 0 | 1386 | 1 | 1005.6625 |
| TCGA-G4-6310 | 0 | 1205 | 1 | 940.8683 |
| TCGA-G4-6310 | 0 | 1205 | 1 | 940.8683 |
| TCGA-G4-6311 | 0 | 1112 | 1 | 986.3589 |
| TCGA-G4-6314 | 0 | 1031 | 2 | 1242.2841 |
| TCGA-G4-6314 | 0 | 1031 | 2 | 1242.2841 |
| TCGA-G4-6315 | 0 | 937 | 2 | 1170.6941 |
| TCGA-G4-6317 | 0 | 894 | 2 | 484.9809 |
| TCGA-G4-6317 | 0 | 894 | 2 | 871.4837 |
| TCGA-G4-6320 | 0 | 678 | 1 | 1030.1 |
| TCGA-G4-6321 | 0 | 572 | 1 | 1859.025 |
| TCGA-G4-6321 | 0 | 572 | 1 | 1859.025 |
| TCGA-G4-6322 | 0 | 547 | 1 | 1561.1627 |
| TCGA-G4-6323 | 0 | 201 | 1 | 1394.8657 |
| TCGA-G4-6586 | 0 | 741 | 1 | 920.034 |
| TCGA-G4-6586 | 0 | 741 | 1 | 920.034 |
| TCGA-G4-6588 | 0 | 644 | 1 | 991.988 |
| TCGA-G4-6588 | 0 | 644 | 1 | 991.988 |
| TCGA-G4-6625 | 0 | 2673 | 1 | 1016.78 |
| TCGA-G4-6625 | 0 | 2673 | 1 | 1016.78 |
| TCGA-G4-6626 | 1 | 1 | 1 | 622.807 |
| TCGA-G4-6627 | 0 | 1434 | 1 | 1718.9064 |
| TCGA-G4-6628 | 0 | 1606 | 1 | 1257.8773 |
| TCGA-G4-6628 | 0 | 1606 | 1 | 1257.8773 |
| TCGA-NH-A50T | 0 | 125 | 1 | 1273.9511 |
| TCGA-NH-A50U | 1 | 334 | 2 | 1762.303 |
| TCGA-NH-A50V | 0 | 154 | 1 | 1317.1179 |
| TCGA-NH-A5IV | 0 | 0 | 1 | 2094.5455 |
| TCGA-NH-A6GA | 0 | 182 | 2 | 1370.5461 |
| TCGA-NH-A6GB | 0 | 170 | 2 | 1049.4132 |
| TCGA-NH-A6GC | 0 | 40 | 2 | 1052.5903 |
| TCGA-NH-A8F7 | 0 | 158 | 2 | 9586.5336 |
| TCGA-NH-A8F7 | 0 | 158 | 2 | 736.5598 |
| TCGA-NH-A8F7 | 0 | 158 | 2 | 9586.5336 |
| TCGA-NH-A8F7 | 0 | 158 | 2 | 736.5598 |
| TCGA-NH-A8F8 | 0 | 167 | 2 | 1131.3869 |
| TCGA-QG-A5YV | 0 | 821 | 1 | 1547.5745 |
| TCGA-QG-A5YW | 0 | 399 | 1 | 1267.2991 |
| TCGA-QG-A5YX | 0 | 526 | 1 | 1922.2586 |
| TCGA-QG-A5Z1 | 0 | 202 | 1 | 1640.784 |
| TCGA-QG-A5Z2 | 0 | 449 | 1 | 1325.6771 |
| TCGA-QL-A97D | 0 | 295 | 2 | 1213.9821 |
| TCGA-RU-A8FL | 0 | 921 | 2 | 855.1317 |
| TCGA-SS-A7HO | 0 | 1829 | 1 | 817.2439 |
| TCGA-T9-A92H | 0 | 0 | 1 | 1329.4068 |
| TCGA-WS-AB45 | 0 | 2038 | 2 | 1271.6356 |
|  |  |  |  |  |
|  |  |  |  |  |
|  |  |  |  |  |
|  |  |  |  |  |
|  |  |  |  |  |
